# Epigenetic Features in Uterine Leiomyosarcoma and Endometrial Stromal Sarcomas: An Overview of the Literature

**DOI:** 10.3390/biomedicines10102567

**Published:** 2022-10-13

**Authors:** Bruna Cristine de Almeida, Laura Gonzalez dos Anjos, Andrey Senos Dobroff, Edmund Chada Baracat, Qiwei Yang, Ayman Al-Hendy, Katia Candido Carvalho

**Affiliations:** 1 Laboratório de Ginecologia Estrutural e Molecular (LIM 58), Disciplina de Ginecologia, Departamento de Obstetricia e Ginecologia, Hospital das Clínicas da Faculdade de Medicina da Universidade de Sao Paulo (HCFMUSP), São Paulo 05403-010, Brazil; 2 UNM Comprehensive Cancer Center (UNMCCC), University of New Mexico, Albuquerque, NM 87131, USA; 3 Division of Molecular Medicine, Department of Internal Medicine, (UNM) School of Medicine, UNM Health Sciences Center, 1 University of New Mexico, Albuquerque, NM 87131, USA; 4 Department of Obstetrics and Gynecology, University of Chicago, Chicago, IL 60637, USA

**Keywords:** uterine leiomyosarcoma, endometrial stromal sarcoma, epigenetics mechanisms, ncRNA, DNA methylation, histones modifications

## Abstract

There is a consensus that epigenetic alterations play a key role in cancer initiation and its biology. Studies evaluating the modification in the DNA methylation and chromatin remodeling patterns, as well as gene regulation profile by non-coding RNAs (ncRNAs) have led to the development of novel therapeutic approaches to treat several tumor types. Indeed, despite clinical and translational challenges, combinatorial therapies employing agents targeting epigenetic modifications with conventional approaches have shown encouraging results. However, for rare neoplasia such as uterine leiomyosarcomas (LMS) and endometrial stromal sarcomas (ESS), treatment options are still limited. LMS has high chromosomal instability and molecular derangements, while ESS can present a specific gene fusion signature. Although they are the most frequent types of “pure” uterine sarcomas, these tumors are difficult to diagnose, have high rates of recurrence, and frequently develop resistance to current treatment options. The challenges involving the management of these tumors arise from the fact that the molecular mechanisms governing their progression have not been entirely elucidated. Hence, to fill this gap and highlight the importance of ongoing and future studies, we have cross-referenced the literature on uterine LMS and ESS and compiled the most relevant epigenetic studies, published between 2009 and 2022.

## 1. Introduction

The body of the uterus is composed of a mucosa muscular interface derived from the Müllerian embryonic ducts and constituted of internal endometrium and external myometrium (MM) tissue layers [1,2,3,4]. The internal endometrium is composed of luminal epithelium, glandular epithelium, and endometrial stroma whereas the MM consists mainly of smooth muscle cells [2,3,4]. Cellular and molecular alterations in the endometrial stroma and smooth muscle cell layers can lead to uterine sarcoma (US) development [4,5,6].

**Figure 1:**
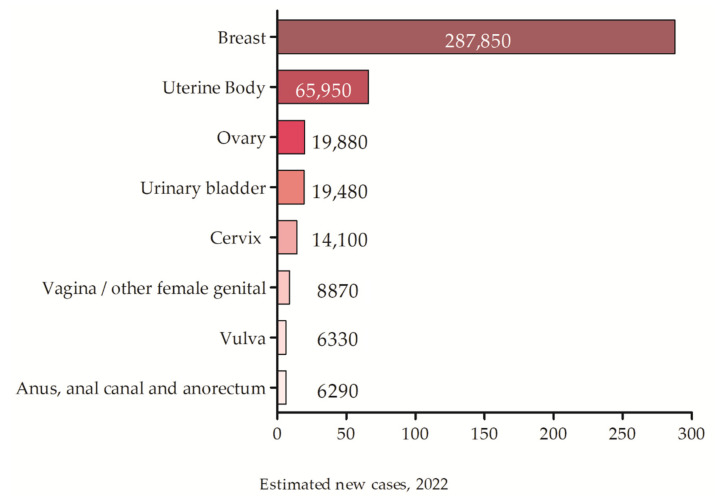
The estimated incidence of gynecological tumors for 2022 according to the ACS.

US accounts for 3–9% of all uterine malignancies and shows high rates of recurrence and metastasis [7,8], occupying the second place among all gynecological tumors [7,9]. The American Cancer Society (ACS) registered a total of 66,570 new cases of uterine tumors with about 12,940 related deaths in 2021 [10] and estimates 65,950 new cases for 2022 (Figure 1) [11].

“Pure” sarcomas are composed exclusively of mesenchymal cells and include the leiomyosarcomas (LMS) and endometrial stromal sarcomas (ESS), which are morphologically classified mainly based on the tumor cells phenotype [12]. LMS arises from the smooth muscle compartment, while ESS arises from the stroma supporting the endometrial glands [8]. LMS and ESS are the most frequent uterine mesenchymal tumors in adult age [13].

For LMS and ESS, the disease stage is the single most important prognostic factor [14]. The International Federation of Gynecology and Obstetrics (FIGO) classification and staging system has been specifically designed for these tumors [15]. In 2018, the ACS published the latest revision of the definitions and clinical staging of LMS and ESS (Table 1), based on the FIGO system and the American Joint Committee on Cancer (AJCC) TNM staging system [14,16,17,18].

It is well known that several molecular events may lead to tumor development. Among these, epigenetic mechanisms such as DNA methylation, post-translational modifications (PTMs), and non-coding RNA (ncRNA) regulation (e.g., microRNAs) can significantly affect the expression of relevant genes, leading to dramatic cell changes [19,20,21]. Epigenetic alterations are characterized by reversibility and susceptibility to external factors and are the main regulatory events governing the development and progression of uterine sarcomas [19,20,22]. Here, we reviewed and summarized the scientific and clinical reports from the past twelve years regarding epigenetic events and their role in the pathophysiology of the ESS and LMS. The most relevant articles written in English were meticulously reviewed and included in this review, and no restrictions for geographic location were applied. Articles without tumor type description or any identification as “uterine” were excluded.

### 1.1. LMS Etiology, Prognosis, and Treatment

LMS arises from the myometrium (MM) and often does not reach the endometrial cavity surface [9,23]. Its incidence is 0.36 per 100,000 women a year, affecting mainly women of ≥40 years of age, and representing approximately 70% of all US [24,25,26,27,28,29]. LMS is a very heterogeneous tumor and represents the most common sarcoma of the uterine body [14,17,25,26,30,31,32]. Its pathogenesis is poorly understood, but several studies focusing on tumor clonality indicate that many of these tumors are de novo entities [33,34,35,36,37,38]. Even though it is an extremely rare event [37], some authors defend the hypothesis that LMSs could arise from the malignant transformation of a pre-existing leiomyoma (LM) [15,30,35,39,40]. However, most of the patients do not exhibit predisposing factors such as prior radiation therapy to the pelvis (10–25%), tamoxifen use (1–2%), genetic syndromes (e.g., retinoblastoma and Li–Fraumeni syndrome), postmenopausal status, and ethnicity (African American) [41].

Clinically, LMS is associated with a poor prognosis even when diagnosed in the early stages, consequently leading to a significant increase in uterine cancer-associated deaths [13,17,26,28,29]. The recurrence rate of LMS reaches 53–75%, even at the initial stages of the disease, with locoregional or distant recurrence in the first two years after diagnosis [7,14,26,28,42,43,44,45,46]. The overall survival expectancy of LMS is 2.6 years, and the survival at 2, 5, and 10 years are approximately 57%, 24%, and 12%, respectively [26,28,42,44]. The survival rates for patients with LMS decrease as the disease progresses; thus, for localized disease (i.e., restricted to the uterus) the estimated survival rates are 64%, for regional disease (afflicting nearby and adjacent tissues, i.e., lymph nodes) the survival rates are 36%, and for disseminated disease [44,47,48,49,50] or metastatic disease (i.e., lungs and liver) the survival is 14% [51].

LMS-related symptoms are associated with vaginal bleeding in 56% of the cases, increased pelvic mass in 54% of the cases, and/or pelvic pain in 22% of the cases [14,15,17,37,39]. Typically, 75% of the patients present a large tumor mass with an average diameter of 10 cm at the time of diagnosis [14,17]. Although LMSs occur primarily in postmenopausal women [52], both progesterone receptor (PR) and estrogen receptor (ER) are found to be expressed in 40% and 70% of the cases, respectively [43,52,53,54,55,56]. A recent review has suggested that hormonal therapy applied to LMS expressing ER/PR is effective and presents favorable tolerance and reliability [57].

There are preoperative methods that allow the differential diagnosis of benign and malignant uterine disease. Magnetic resonance imaging (MRI) remains the optimal imaging modality to characterize pelvic masses originating from the uterus, but distinguishing LMS from LM remains a challenge [17].

LMS histopathological analysis is characterized by the presence of spindle cells, with ruptured nuclei, perinuclear vacuolization, and eosinophilic cytoplasm arranged in intersecting fascicles within the analyzed sample. Meeting the Stanford criteria, LMS should be deemed intrinsically as a high-grade tumor [58]. Cell atypia can vary from moderate to severe, while nuclear atypia is always severe, large areas of tumor cell necrosis with variable mitotic index and atypical mitosis are often observed [15,24,37,59,60]. There are two uncommon subtypes of uterine LMS: myxoid and epithelioid LMS. These present mild or focal nuclear pleomorphism and lower mitotic degree, compared to typical LMS. Diagnostic mistakes between these types of LMS and other smooth muscle tumors are often common [61,62].

Immunohistochemical co-expression of Desmin, h-Caldesmon, smooth muscle actin (SMA), and HDAC8 can assist with LMS diagnosis [39,40,56,63,64,65]. Several other potential biomarkers such as PDGFRA, WT1, GNRHR, BCL2, ESR, PGR, and LAMP2 have also been evaluated to distinguish LMS from other tumors, mainly from LM [63]. The cell proliferative index (determined by Ki-67 protein expression), the protein expression levels of the tumor suppressors p16 and p53, and the expression of several isoforms of the CD44 (hyaluronan receptor) are, however, routinely used [8,65]. Additionally, some patients show high amounts of lactate dehydrogenase (LDH) [12] and/or CA125 levels [13], but these markers are quite unspecific [17].

Most recently, gene expression profile analysis has enabled the classification of LMS into two subtypes according to their molecular signature. The subtype I recapitulated the low-grade LMS and was enriched for *LMOD1*, *SLMAP*, *MYLK*, and *MYH11*, all of them smooth muscle-specific markers. Subtype II of LMS included tumors with worse prognosis and expressed genes associated with cell cycle, proliferation, and tumorigenesis (*CDK6*, *BMP1*, *MAPK13*, *PDGFRL,* and *HOXA1*) [66,67].

The gold standard treatment for LMS is still the tumor surgical excision. Total hysterectomy and bilateral salpingo-oophorectomy are recommended for early-stage tumors [17,32,46,47,48,56,58,68]. Adjuvant chemo and radiotherapies are indicated to avoid recurrences, or for early-stage disease, but their effectiveness is still unclear [17,44,49,69] and they do not offer a significant advantage to improve overall survival [22,43,49,55,70,71]. Recently, new chemotherapies, targeted therapies (pazopanib), and immunotherapies (nivolumab or pembrolizumab) seem to be promising new approaches to treat drug-resistant LMS [41].

### 1.2. ESS Etiology, Prognosis, and Treatment

ESS is the second most common type of US [72] and arises from the uterine stroma. It is composed of endometrial stromal cells reminiscent of proliferative phase endometrium [7,13,15,73]. It is predominantly intramural, showing both a myometrial invasion and myometrial lymphovascular space permeation [7]. ESS pathogenesis is unknown, but tamoxifen exposure and some medical conditions (e.g., polycystic ovary syndrome) may contribute to its development [73,74]. Although a rare tumor, representing less than 1% of all uterine tumors, ESS accounts for up to 25% of all uterine sarcomas [58]. Symptoms related to ESS development and progression include abnormal uterine bleeding (about 90% of patients), uterine enlargement (70% of cases), pelvic pain, and dysmenorrhea. In 25% of the cases, however, the patients can be asymptomatic [73,75].

The most recent World Health Organization (WHO) classification (2020) for ESS is based on both cytogenetic and molecular analyses (i.e., gene fusion or alterations [76]) where the tumors are divided into benign endometrial stromal nodules (ESN), low-grade endometrial stromal sarcoma (LG-ESS), high-grade endometrial stromal sarcoma (HG-ESS), and undifferentiated uterine sarcomas (UUS) (Table 2) [76,77,78]. Morphologically, ESN is differentiable from LG-ESS only for the absence of lymphovascular invasion and myometrial infiltration. LG-ESS is usually positive for CD10, ER, and PR and can express actin, keratins, and calretinin [79], which differ from the HG–ESS tumors carrying the *YWAE-NUTM2* fusion that do not express these markers. HG-ESS shows high expression of Cyclin-D1, c-KIT, and BCOR, and when *ZC3H7B-BCOR* fusion is present, CD10 and variable staining for ER and PR are also observed. Finally, UUS exhibits myometrial invasion, severe nuclear pleomorphism, high mitotic activity and/or necrosis, and loss of differentiation. This tumor, however, does not show a specific immunohistochemical profile, instead showing a diffused and atypical staining for CD10 as well as heterogeneous patterns of ER and PR staining [14,80].

HG-ESS and UUS can be difficult to diagnostically differentiate from LMS since the latter can mimic both ESS and UUS. In this case, the immunohistochemical markers and morphological features can be useful, but not accurate. Tumor location can also provide important information because LMS is exclusively related to MM, while UUS may also involve the endometrium. Furthermore, ESS is also diagnosed only post-surgery, but unlike LMS, they present an indolent course with relapses occurring up to 20 years after diagnosis [81].

Overall, patients with ESS have a better life expectancy than other sarcomas. Their five-year survival rates are higher than 80%. For disease stages I and II the five-year survival is approximately 90% whereas for stages III and IV (i.e., advanced disease) the survival rate for the same interval of time is significantly reduced, according to the FIGO stage system [75,82].

Treatment protocols are defined based on the grade and stage of the tumor at the time of diagnosis. Total hysterectomy with bilateral salpingo-oophorectomy remains the standard treatment for ESS, and lymphadenectomy does not appear to improve overall survival rates [83]. Adjuvant radiotherapy and hormone therapy are not well-established therapeutic options yet, even though some studies have shown that hormonal agents can be an alternative to the management of LG-ESS [82,84]. In contrast, HG-ESS is generally detected in advanced stages with no effective adjuvant therapy available. In this case, immunotherapy with adoptive T cells transfer, targeting tumor fusion proteins, can be useful. Such an approach has been proved to be efficient in inhibiting tumor recurrences in other cancer types, thus inducing long-term memory cells and the persistent presence of these cells in the patient’s blood [85].

## 2. Genetics and Epigenetics Mechanisms in LMS and ESS

**Figure 2:**
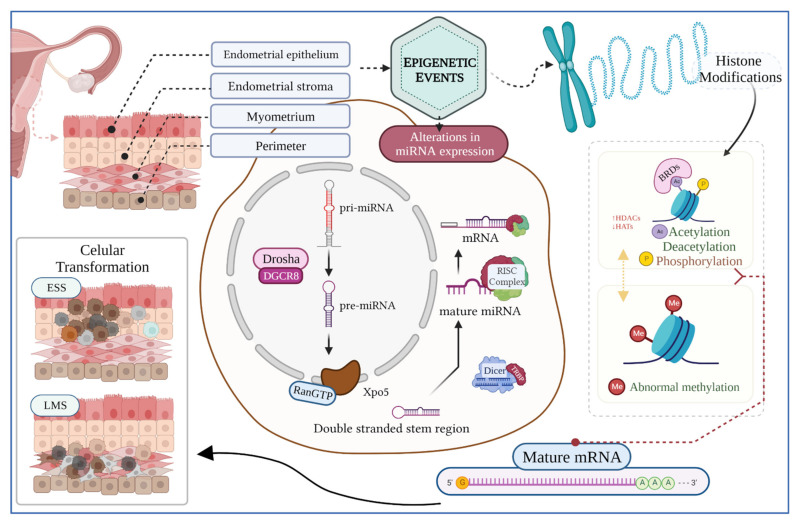
Graphical representation of epigenetic events potentially involved in the initiation and development of the tumors, including uterine LMS and ESS. The biogenesis of miRNAs starts in the nucleus and ends in the cytoplasm. This process includes the participation of several enzymes and protein complexes that regulate the production of mature molecules capable of regulating gene expression, both through induction of mRNA degradation and translational repression. Likewise, dynamic alterations of histone modifications, including acetylation, methylation, and phosphorylation, modify gene expression, thus affecting DNA replication and repair, chromatin compaction, and cell cycle control. In addition, histone modification readers such as BRDs can recognize modified histones, therefore altering gene expression and responding to different signals. Dysregulation in the epigenetic machinery leads to malignant transformation of cells culminating in the development of cancer. Created with BioRender.com.

Genetic changes are related to alterations in the DNA sequences, whereas epigenetic modifications involve specific regulatory events apart from DNA codification [102,103,104,105,106,107,108]. Epigenetic events play an important role in several normal cellular processes, including embryonic development, genetic imprinting, and X-chromosome inactivation. When altered, epigenetic mechanisms may lead to several diseases, including cancer initiation and progression [106]. Epigenetic dysregulation affects gene functions by altering the gene expression mainly by (1) DNA methylation, (2) PTMs, and (3) RNA-mediated gene silencing by ncRNA (e.g., microRNA) (Figure 2) [102,109]. The main clinical and scientific interest in epigenetic events resides in the fact that they are reversible mechanisms [110,111].

### 2.1. DNA Methylation

DNA methylation is the most studied and understood epigenetic event described to date. Found in more than 70% of the human genome, DNA methylation is crucial for cellular differentiation and normal development [112]. It consists of the addition of a methyl radical (CH3) to the 5-carbon on cytosine residues (5mC) in CpG dinucleotides [103,104,108,109,110,111,112,113,114]. DNA methyltransferases (DNMTs)—enzymes responsible for DNA methylation—are known to act in cancer cells by either hypomethylation or hypermethylation of specific CpG regions in the DNA [114].

**Figure 3:**
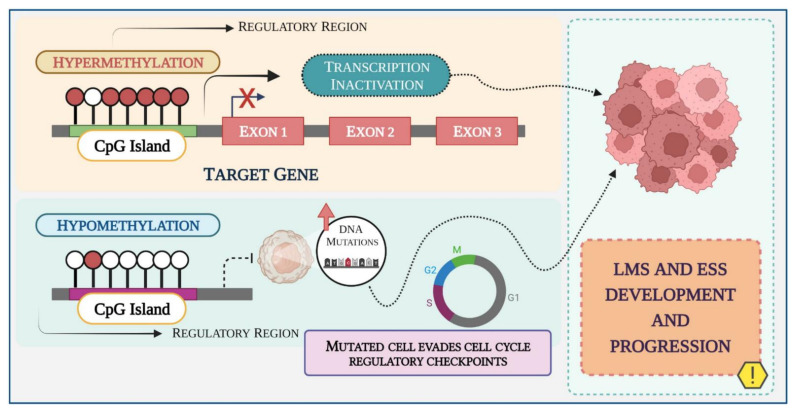
Schematic representation of the DNA methylation process in LMS and ESS during cancer development and progression. Methyl groups are added to the DNA molecule and change its activity. Promoter hypermethylation has been shown to silence tumor suppressor genes in cancer cells, leading to either dysregulation of cell growth or inducing resistance to cancer therapies. Hypomethylation promotes genomic instability causing missegregation of chromosomes during cell division. Created with BioRender.com.

Global DNA hypomethylation or loss of methylation has been associated with genomic instability as well as aneuploidy, loss of imprinting, reactivation of transposable elements, and endogenous retrovirus (ERVs) [108,113,115]. In cancer, hypomethylation is commonly followed by hypermethylation of localized CpG islands at the promoter and regulatory regions of target genes, which remain unmethylated in normal cells [109,115,116]. Hypermethylation of regulatory regions leads to transcriptional silencing by directly blocking the transcription factors binding to the promoter region, or by the binding of proteins with a high affinity for methylated DNA that compete with the transcription factors binding sites (Figure 3) [108,117].

DNMTs are commonly found overexpressed in tumors, constituting an attractive target for specific therapy. The FDA has approved “epidrugs” such as 5-azacitidine (5-Aza), 5-aza-2-deoxycytidine (DAC), and the second-generation of the demethylation agent guadecitabine [116,118]. In LMS, Fischer et al. (2018) assessed the therapeutic potential of nucleoside analogs 5-Aza, DAC, and guadecitabine, using both in vitro and in vivo experiments. Their results show guadecitabine as a more effective inhibitor of both cell survival and colony formation in vitro. Additionally, animals who received this treatment showed a decrease in the tumor burden and increased survival [116].

Our group recently assessed the impact of DNMT inhibition on the Hedgehog (HH) signaling pathway with 5′-Aza-dc, in uterine LMS cells. We observed a reduction in the *GLI1* mRNA, and SMO and GLI1 protein in response to the treatment. Moreover, GLI1 and GLI2 nuclear translocation were also decreased while nuclear translocation of GLI3 was increased. Our data showed that DNMT inhibitor, alone or in combination with pharmacological treatment, was able to block the HH pathway and showed a high inhibitory effect on the LMS malignant cells phenotype [119].

*MGMT* silencing due to its promoter hypermethylation has been commonly observed in several malignancies, including uterine sarcomas [120,121]. The methylation of the *MGMT* promoter region, which contributes to genome instability and sensitizes the cells to alkylating agents (such as Temozolomide (TMZ)), has been correlated with improved prognosis and as a potential factor of response to TMZ-based therapy prediction [121].

Global DNA methylation studies have also found methylation patterns or signatures that have been associated with different cancer hallmarks [122]. Braný et al. in 2019 observed differences in the methylation levels between MM and LM samples in the *KLF4* and *DLEC1* genes. Higher levels of methylation were found in LM compared to LMS cases, suggesting that methylation of *KLF4* and *DLEC1* are potential biomarkers to distinguish LM from LMS [123].

Hasan et al. (2021) identified differentially methylated and differentially expressed genes associated with LMS. Among the 77 hypermethylated genes, chromatin-modifying enzymes, including KAT6A, KMT2A and EZH2, and chromatin/DNA binding proteins such as CTNNB1, PBX3, SATB1, MEIS and COMMD1-BMI1 were observed. The findings indicate the possible involvement of chromatin modulation in regulating the DNA methylation of these genes [124].

A higher DNA damage response and hypomethylation of *estrogen receptor 1* (*ESR1*) target genes were both observed when comparing uterine to extra uterine LMS [13].

Gene silencing through methylation can occur as frequently as mutations or deletions, leading to aberrant silencing of tumor suppressor genes [125]. In an LMS experimental model, the lack of *BRCA1* function was associated with tumor initiation and development. This protein expression was next investigated in human samples, and a loss of 29% was associated with promoter methylation [126]. Methylation in the *CDKN2A* gene, using uterine LMS samples with a rhabdomyosarcomatous component, has also been described [127]. The authors identified both methylated and unmethylated alleles, originating mainly from the smooth muscle component. Moreover, the loss of heterozygosity in the rhabdomyosarcomatous component has been described exclusively in the cells expressing p16 and p14 [127].

Hierarchical clustering based on the hypermethylation of ALX1, *CBLN1*, *CORIN*, *DUSP6*, *FOXP1*, *GATA2, IGLON5, NPTX2*, *NTRK2*, *STEAP4*, *PART1*, and *PRL* allowed differentiation among several uterine tumors with up to 70% accuracy [128,129]. Clusters of distinct DNA methylation patterns have also been useful in distinguishing tumor types regardless of the number of CpG sites. Thus, LM and LMS were separated into two different clusters each, while ESS samples (LG- and HG-ESS) were grouped into two subtypes with specific profiles. The HG-ESS cluster included *YWHAE* and *BCOR*-rearranged tumors, distinct from LG-ESS and LMS [128,129]. Although LMS and HG-ESS are morphologically similar, the results show that the DNA methylation profile may be useful to discriminate against these closely related tumors [128].

DNA methylation plays a key role in gene expression regulation, inducing functional changes in key genes that regulate endometrial homeostasis [113]. Li et al. (2017) showed that the *KLF4* promoter was hypermethylated in the ESS. They also found *PCDHGC5* was highly methylated in the ESS samples when compared to endometrioid and endometrial serous carcinoma. *sFRPs 1-5* are tumor suppressors that downregulate Wnt/β-catenin signaling [130,131,132,133]. Their consistent promoter hypermethylation and subsequent gene expression suppression were described in ESN, LG-ESS, and UUS.

Although the true role of DNA methylation patterns in the LMS and ESS initiation and progression is not completely understood, DNA methylation in normal endometrial stromal cells has been useful to identify signatures that indicate changes during decidualization or cell transformations [134,135,136,137]. Further studies to determine the precise methylation profile in pure mesenchymal tumors are certainly necessary to enable the characterization and differentiation of these tumors as well as to establish new therapeutic options.

### 2.2. Chromatin Remodeling

The chromatin is composed of DNA molecules tightly coiled around proteins called histones. This structure condensation degree is directly associated with greater or lesser RNA synthesis, with greater condensation (higher chromatin closing) being the state of more transcriptional repression [138]. The basic unit of chromatin, called nucleosome, is constituted by two copies of each core histone (H2A, H2B, H3, and H4) enveloped by DNA molecules [139]. Chromatin regulation occurs through PTMs of core histones and can involve phosphorylation, acetylation, methylation, ubiquitination, SUMOylation, and GlcNAcylation [139]. 

Currently, the two most studied and best understood mechanisms of chromatin regulation are histones acetylation and methylation. Such events are regulated by very specialized proteins called writers, erasers, and readers—which respectively add, remove, or recognize these PTMs [110,111,112,140,141,142]. Included in the writers’ group are histone acetyltransferases (HATs), DNA methyltransferases (DNMTs), histone lysine methyltransferases (HKMTs), and histone methyltransferases (HMTs). The erasers’ group includes histone deacetylase (HDACs) and histone demethylases (HDMs), ten eleven translocations (TETs), and histone lysine demethylases (HKDMs). In the reader’s group are methyl-CpG-binding domains (MBDs) and bromodomains [143].

**Figure 4:**
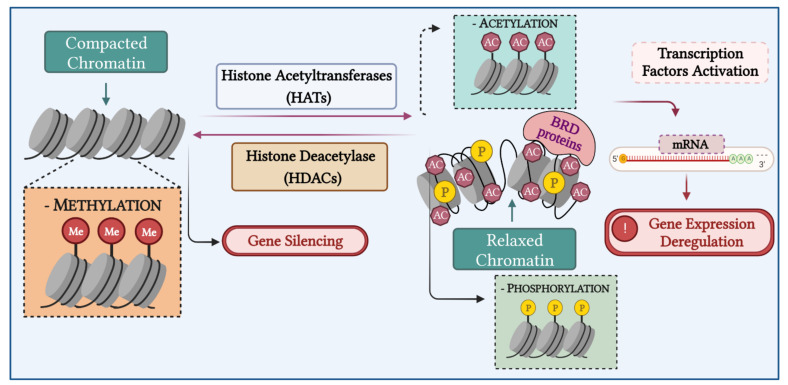
Chromatin remodeling process in LMS and ESS. Chromatin remodeling is an important mechanism of gene expression regulation. In the histone acetylation induced by HATs, the condensed chromatin is transformed into a more relaxed structure (euchromatin) that is associated with greater levels of gene transcription, while histone hypoacetylation induced by HDAC activity is associated with more condensed chromatin (heterochromatin), inducing gene silencing. Altered expression and mutations of genes that encode HDACs have been linked to tumor development. Created with BioRender.com.

Histone modifications affect the chromatin structure providing binding sites for several transcriptional factors. Its modification has a direct influence on gene expression, DNA replication and repair, chromatin compaction, and cell cycle control. Thus, loss of regulation of the histone modifications can lead to cancer pathogenesis and several developmental defects (Figure 4) [143].

The lysine residues of histones H3 and H4 are targeted for methylation by site-specific enzymes, culminating in activation or repression of the gene expression. [144]. This molecular mechanism is uniquely able to originate three methylation levels: me1 (mono), me2 (di), and me3 (trimethylation). Lysine methylations may lead to both transcriptional activation and repression, depending on the lysine residue’s location [143].

In endometrial stromal cells, the transition from a proliferative to a decidual phenotype occurs due to the loss of the EZH2-dependent methyltransferase activity, which is part of the chromatin remodeling process [145]. The decidualization process in those cells down-regulates EZH2, resulting in lower levels of H3K27me3 at the promoter region of *PRL* and *IGFBP1* (two decidual marker genes). The H3K27me3 loss, associated with acetylation enrichment in the same lysine residue, indicates the transition from a transcriptionally repressive chromatin form to a permissive one [145].

Little is known about the specific underlying mechanism of histone acetylation or methylation associated with the “pure” sarcoma pathobiology. A unique study available in the literature describes the fatty acid synthase (FASN)-enhanced expression inducing cell proliferation, migration, and invasion, in transfected cells of uterine LMS. It has been observed that FASN promotes H3K9me3 and H3K27ac by alteration in the HDAC, HDM, HMT, and HAT trimethylation activity. Thus, in the uterine LMS cells, the epigenome reprogramming by chromatin remodeling seems to induce a higher malignant phenotype [146].

The polycomb group (PcG) proteins are well-characterized transcriptional repressors that are essential for the regulation of physiological processes in several organisms. PcG proteins are known to form two distinct complexes with defined enzymatic activities: polycomb repressive complex 1 (*PRC1*), a histone ubiquitin ligase related to chromatin compaction; and *PRC2*, an *HMT* that mediates both H3K27me3 and target genes repression [147,148,149,150]. In several cancer types, the expression and function of PcG proteins are often found dysregulated [148,151,152,153], and their targeted deletions generally induce lethal phenotypes [153].

*PRC1* catalytic core has two related E3 ubiquitin ligases, the *RING1* (RING1A) or *RNF2* (RING1B) that catalyze ubiquitination and BMI1 (polycomb ring finger oncogene), and one of six *PCGF* orthologues. The latter constitutes a *PRC1* variant containing *BCOR* and *KDM2B* [154,155,156,157]. Translocations or chromosomal rearrangements, involving fusion proteins have also been implicated in PRCs mechanisms. Fusions such as *KDM2B-CREBBP* [158], *ZC3H7-BCOR* [79,155,156,159,160], *JAZF1-BCORL1* [79,91,161], *EPC1-BCOR* [72,95], *LPP-BCOR* [72] and *BCOR* internal tandem duplications (*BCOR-ITD*) are frequently found in ESS, and have recently been also found in LMS samples [80,160,162,163]. Additionally, gene fusion such as *MBTD1-CXorf67* [79,91,151,164], *MBTD1-EZHIP* [90] and *MBTD1-PHF1* in ESS are found as products of PRC1-associated protein [152].

*PRC2* has in its core subunits the *EZH2* (or its homolog *EZH1)*, *EED*, *SUZ12*, and *RbAp46* (or *48*). *EZH1* and *EZH2* are responsible for the H3K27me3 generation. *EED* binds to H3K27me3, enhancing the *EZH2* catalytic activity, while *SUZ12* is essential for the *PRC2* activity, and bAp46/48 acts as a chaperone. H3K27 trimethylation leads to the suppression of several relevant genes, including tumor suppressors [165].

There are two well-characterized PcG proteins, the BMI1 and EZH2, which are required for the regulation of the PRC activity but are also known to display oncogenic functions in several cancers. BMI1 was the first identified PcG protein that was described as a proto-oncogene, and although there are no specific studies regarding the BMI1 role in uterine LMS [157,166]. Gao et al. (2021) have described one CD133 cell subpopulation that was derived from SK-UT-1 (uterine LMS cells) with enhanced levels of this protein. The authors found BMI1, among other CSCs-related (cancers stem cell) markers, up regulated in the CD133^+^ cells when compared to the negative cell population [167].

Zhang et al. (2018) investigated both gene and protein expressions of the four PRC2 subunits (*EZH2*, *SUZ12, EED,* and *RbAp46*) in extra-uterine and uterine LMS samples. The authors observed 91% of sensitivity and 100% of specificity for *EZH2* positive staining in well-differentiated LMS, suggesting this expression is a specific marker for this tumor. Furthermore, the increased expression of EZH2 was inversely correlated with *SUZ12* and *EED* expressions, leading to *PRC2* suppression and H3K27me3 decrease [168].

Chromosomal rearrangements in genes belonging to the PRC2 complex, or in proteins that interact with them, have previously been described in the ESS [131]. *JAZF1-SUZ12* (previously named *JAZF1-JJAZ1*) has been frequently reported as the most common feature of ESS [131,164,169,170,171,172]. Additionally, several other modifications such as *EPC1-PHF1* [158,172,173], *MEAF6-PHF1* [87,161,162,170], *EPC1-ZUZ12* [72,95], *MEAF6-SUZ12* [91,174], *BRD8-PHF1* [169], *JAZF1-PHF1* [88,158] and *YWHAE-NUTM2A/B* (previously known as *FAM22A/B*) [79,132,175] have also been reported. Panagopoulos et al. in 2012 observed that the rearrangement of genes involved in acetylation and methylation can be associated with ESS pathogenesis. LG-ESS harboring the *EPC1-PHF1* fusion gene has decreased levels of H3K27me3 and a concomitant increase of H3K36me3 [176]. *PHF1* acts in cell proliferation through the modulation of histone H3 methylation [171].

*EZH2* can interact with HDAC1 and HDAC2, through the EED protein, suggesting that the transcriptional repression by the PRC2 complex may be mediated by *HDACs* [177]. These enzymes act in the acetylation control of transcription factors [178], and their classification (Class I, IIa, IIb, III, and IV) is based on their activity, structural similarity, subcellular localization, and expression patterns [179]. In LMS patients, strong expression of HDACs 1, 2, 3, 4, 6, and 8 were associated with unfavorable prognosis, while HDACs 5, 7, or 9 weak expressions, together with p53 expression, were associated with favorable disease-free survival (DFS). HDACs 5, 7, and 9 were associated with better survival outcomes, whereas HDAC5 expression was an independent predictor for DFS in epithelioid subtype tumors [180]. In vitro analysis using SK-UT-1, SK-LMS-1, MES-SA, and DMR cell lines demonstrated that HDAC9 (Class IIa) transcription is under *MEF2D* direct control, and this axis sustains cell proliferation and survival through *FAS* repression [177].

In ESS, it has been observed that high expression of HDACs 1, 4, 6, 7, and 8 is associated with lower DFS [181] whereas, in UUS, distant tumor recurrence was associated with a strong expression of HDAC6 [140,182].

The increased HDAC activity often observed in cancers justifies the number of current studies investigating HDAC inhibitors as novel therapeutic agents [183,184]. These studies have shown promising results for metastatic LMS [140,180,181,185]. In this context, mocetinostat acts by turning on tumor suppressor genes, restoring their normal function, and reducing tumor growth [140,180]. Its use as mono- or combinatorial therapy has been evaluated in metastatic extra-uterine and uterine LMS with resistance to gemcitabine and found to induce regression of tumors with acquired chemoresistance. Romidepsin, LBH589, belinostat, SAHA, and valproate (other HDAC inhibitors) have shown good results alone or in combination with decitabine [180,186].

Combinatorial therapy using SAHA, LY294002 (PI3K inhibitor), and rapamycin (mTOR inhibitor) were tested in ESS cell culture [186]. The results show that SAHA combined with either LY294002 or rapamycin, or both, reduce specifically phospho-p70S6K and 4E-BP1 levels, inhibiting the tumor cell proliferation [186,187]. A strong reduction of mTOR and phospho-mTOR levels has been reported after treatment with either SAHA or rapamycin, by targeting phospho-S6rp, in ESS cells [188]. Fröhlich et al. (2014) showed the benefit of SAHA treatment associated with TRAIL/Apo-2L in two US cell lines [189]. 

Another study assessed the effects of combined therapy with valproic acid (VPA, a weak histone deacetylase inhibitor), bevacizumab (mAb against VEGF), gemcitabine, and docetaxel, for extra- and intrauterine unresectable or metastatic soft tissue sarcomas [184]. This study found partial response in one case of carcinosarcoma, two extrauterine LMSs, two undifferentiated pleomorphic sarcomas, and one uterine LMS patient. This pharmacological combination was well tolerated and overall safe, showing that the combination of traditional medication and “epidrugs” may truly represent a new treatment strategy for sarcoma [184].

New therapeutic strategies to specifically treat US, such as regional hyperthermia, combined with chemotherapy, radiotherapy, and/or immunotherapy have emerged in the last few years. Pazopanib (a multitargeted tyrosine kinase inhibitor with antiangiogenic effects) combined with hyperthermia has demonstrated synergistic effects mainly for LMS growth inhibition, in vitro and in vivo [185]. This approach induces *HAT1* downregulation by suppressing Clock which, in turn, is responsible for H3 and H4 acetylation [190].

Histone phosphorylation, which takes place predominantly but not exclusively on serine, threonine, and tyrosine residues at the histone tails [142], has gained considerable attention, especially regarding the histone H3, due to its close association with mitotic chromosome condensation in mammalian cells [191]. A preliminary study evaluating the mitotic index, based on H3 phosphorylation in LMS, found Phospho Histone H3 (PHH3) positive staining to be a promising mitosis-specific marker for this tumor [192].

Bromodomain-containing proteins (BRDs), as the “readers” of lysine acetylation are responsible for transducing regulatory signals carried by acetylated lysine residues into various biological phenotypes [193]. BRDs can exert a wide variety of functions via multiple gene regulatory mechanisms [194] and the deregulation of BRDs is involved in many diseases, including cancer [195,196,197]. BRD9 is a newly identified subunit of the noncanonical barrier-to-autointegration factor (ncBAF) complex and a member of the bromodomain family IV [198]. Studies have demonstrated that BRD9 plays an oncogenic role in multiple cancer types, by regulating tumor cell growth. Furthermore, the connection of BRD9 with the PI3K pathway [199], microRNAs [200], and STAT5 [201] is implicated in cancer progression. It has been shown that BRD9 is aberrantly overexpressed in uterine LMS tissues, compared to adjacent myometrium. [202]. In addition, BRD9 expression was upregulated in uterine LMS cell lines compared to benign LM and myometrium cell lines. Notably, targeting the BRD9 with a specific chemical inhibitor (TP-472) can suppress the LMS cell growth, concomitantly sculpting the transcriptome of uterine LMS cells, altering the important pathways, reprogramming the oncogenic epigenome, and inducing the miRNA-mediated gene regulation. These studies reveal that BRD9 constitutes a specific vulnerability in malignant LMS and that targeting non-bromodomain and extra-terminal BRDs in uterine LMS may provide a promising and novel strategy for treating patients with this aggressive uterine cancer [202].

In summary, histone modifications are frequently found in US, thus representing potential targets for new therapeutic strategies development. Several studies have demonstrated that HDAC inhibitors could modulate several signaling pathways, activating, or inhibiting numerous cascades that lead to an antitumor response. Moreover, combination with chemo- or targeted- therapies is likely to strengthen the activity of HDAC inhibitors. However, it is still necessary to further elucidate how the histone modifications are regulated, as well as to understand the mechanism of action of their available inhibitors in LMS and ESS. This information will enable more efficient clinical trials, which could lead to an improvement in patients’ response to treatment and overall survival [140].

### 2.3. Non-Coding RNA (ncRNAs)

**Figure 5:**
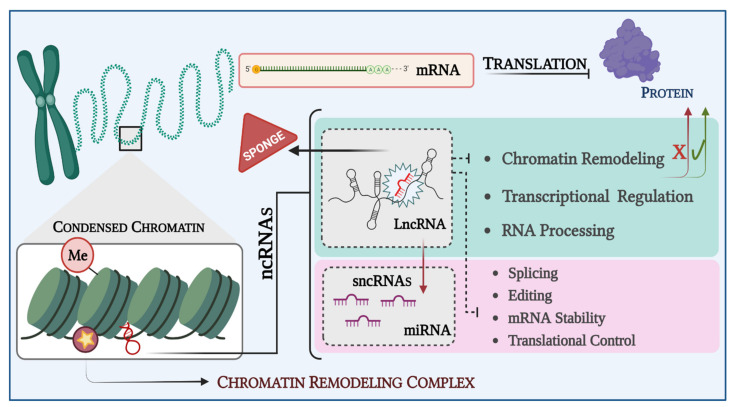
Graphical representation of the ncRNAs dysregulation in LMS and ESS. ncRNAs have been identified as oncogenic drivers or tumor suppressors in cancer. LncRNAs often affect the expression of their target genes by interacting with miRNAs, which are the main post-transcriptional regulation factors. Some lncRNAs act like sponges, thereby preventing miRNAs from binding to their target mRNAs. As lncRNAs work as decoys for miRNAs, oncogene mRNA translation is allowed, starting the LMS and ESS carcinogenesis. Created with BioRender.com.

ncRNA is known to regulate gene expression both at transcriptional and post-transcriptional levels. ncRNAs play an important role in epigenetic processes, including modulation of heterochromatin, histone modification, DNA methylation, and gene silencing (Figure 5) [108]. These molecules can be divided into housekeeping and regulatory ncRNAs [110,203]. Regulatory ncRNAs are classified according to their size in small non-coding RNAs (sncRNAs), with approximately 19-200 nucleotides (nt), and long non-coding RNAs (lncRNAs), with more than 200 nt [105,108,113,204,205]. The sncRNAs have a wide range of structural and functional roles in gene expression regulation, RNA splicing, and chromatin structure [206,207]. sncRNAs includes four different categories: (1) small interfering RNA (siRNA), (2) microRNA (miRNA), (3) PIWI-interfering RNA (piRNA, with approximately 19-31 nt), and (4) small nucleolar RNA (snoRNA, with 60-300 nt) [111,203]. miRNA and piRNA are probably the most studied sncRNAs categories to date, and their functions are well established in the literature [206]. Due to miRNAs’ broad roles, mainly at the post-transcriptional level, dysfunctions in their regulation have been associated with the development of several diseases, including cancer [205,206,208]. 

To understand the complex biology of sarcomas, numerous correlative and functional studies aiming to integrate gene expression patterns and miRNAs have been carried out [209]. As mentioned above, the differential diagnosis of LMS is still a challenge, and studies focusing on new biomarkers to help distinguish uterine LMS from LM are extremely important [210,211,212,213]. Yokoi et al. (2019) demonstrate the feasibility of circulating serum miRNAs detection as a preoperative clinical assay to detect US. They identified two miRNA signatures (*miR-1246* and *miR-191-5p*) in uterine LMS (95% confidence interval of 0.91–1.00) [214]. Dvorská et al. (2019), and later, Wei et al. (2020) reviewed how liquid biopsies could increase the overall understanding of uterine LMS behavior and how its molecular profile could contribute to more accurate discrimination from LM [210,211].

Comparing LMS, LM, and MM, Anderson et al. (2014) found 37 miRNAs differentially expressed in uterine LMS. The lack of *miR-10b* in LMS samples was critical for tumor growth and metastasis. Indeed, rescuing *miR-10b* expression in the cell lines resulted in prominent inhibition of cell proliferation, migration, and invasion, and increased apoptosis. Similarly, stable *miR-10b* expression significantly reduced the number and size of tumor implants in vivo by reducing cell proliferation and increasing apoptosis [212].

Later, Schiavon et al. (2019) found that dysregulation of *miR-148a-3p*, *27b-3p*, *124-3p*, *183-5p,* and *135b-5p* expression was associated with tumor relapse, increased metastasis, and poor survival rates in uterine LMS patients [213]. De Almeida et al. (2017) evaluated the miRNAs expression profile in cell lines of MM, LM, and LMS. Thirteen molecules presented differential expression profiles in LM and LMS, compared to normal tissue (MM). Additionally, the authors observed that *miR-1-3p*, *miR-130b-3p*, *miR-140-5p, miR-202*, *miR-205*, and *miR-7-5p* presented similar expression patterns between the cell lines and 16 patients’ samples [215].

Zhang et al. (2014) demonstrated that miRNAs were significantly dysregulated among different types of uterine smooth muscle tumors (USMTs), including ordinary LM, mitotically active leiomyoma (MALM), cellular leiomyoma (CLM), atypical leiomyoma (ALM), uterine smooth muscle tumor of uncertain malignant potential (STUMP), and LMS samples. The miRNA expression profile showed that ALM and LMS shared similar signatures (including *miR-34a-5p*, *miR-10b-5p*, *miR-21-5p*, *miR-490-3p*, *miR-26a-5p* and *miR-650)*. Unsupervised analysis divided the tumors into three clusters: LMS/ALM, LM/STUMP, and CLM/MM [216]. *miR-200c* was found to be significantly downregulated in LM, compared to MM [217], acting directly in *ZEB1/2*, *VEGFA*, *FBLNS,* and *TIMP2* regulation. Next, the authors observed a significant reduction of *miR-200c* in the SK-LMS-1 cells, compared to isolated LM cells, indicating this miRNA is an important marker for LM progression and malignance risk [4,218].

To date, the differential expression (i.e., up- or down-regulation) of several miRNAs has been directly correlated with US patients’ prognosis. In 2018, Dos Anjos et al. analyzed the expression profile of 84 cancer-related miRNAs and associated their signatures with patients’ clinical and pathological data. In LMS, specifically, the authors found an association between miRNA dysregulation and lower cancer-specific survival (CSS) and aggressive tumor phenotype. In ESS samples, alterations in miRNA regulation were related to both lower CSS and metastasis [219].

Shi et al. (2009) found a significant inverse correlation between endogenous *HMGA2* levels and *let-7* expression in uterine LMS. Their study revealed that the ectopic expression of *let-7a* inhibits LMS proliferation by *HMGA2* repression, suggesting that the *let-7* loss of expression can represent a worse prognostic factor [220]. Zavadil et al. (2010) identified the way in which let-7s is responsible for the direct regulation of PPP1R12B, *STARD13*, *TRIB1*, *BTG2, HMGA2,* and *ITGB3* genes (involved in the cell proliferation and extracellular matrix regulation) in LM samples. [221]. De Almeida et al. (2019), found that decreased expression of *let-7* family members was directly correlated with worse prognosis, affecting both the overall survival (OS) and the DFS rates of the LMS patients [22].

Dysregulation of some miRNAs has also been correlated with acquired chemoresistance in uterine neoplasm. For instance, the loss of *miR-34a* expression and its release from LMS cells via exosomes contribute indirectly to the tumor doxorubicin chemoresistance. This mechanism seems to be mediated by *MELK* overexpression and the recruitment of M2 macrophages [216].

Although less studied than other ncRNAs, lncRNAs are known to interact with either DNA, RNA, or proteins, and play a significant regulatory function in several cellular processes [208]. lncRNAs are responsible for regulating transcription on three different levels: pre-transcriptional (chromatin remodeling), transcriptional, and post-transcriptional [203,222]. Some similarities can be found between lncRNAs and mRNAs, including size, transcription by RNA pol-II, 5′-capping, RNA splicing, and poly(A) tail (approximately 60% of all lncRNAs) [223]. LncRNAs can be stratified into five categories: (1) intergenic (present between two protein-coding genes), (2) intronic (between the introns of a protein-coding gene), (3) overlapping (a coding gene is located on the intron of a lncRNA), (4) antisense (the lncRNA is transcribed from the opposite strand of a protein-coding gene), and (5) processed lncRNAs (lacks an open reading frame ORF) [208,224]. lncRNA can be expressed in distinguished cell regions and their functions are directly related to their sub-cellular location. However, these epigenetic regulators may suffer molecular alterations that affect their expression and, consequently, their physiological function. Accumulated evidence shows that several differentially expressed lncRNAs are related to cancer development, progression, and metastasis [204,222].

Unfortunately, in uterine LMS and ESS, the molecular role of lncRNAs and their regulation remains unclear. Yet, Guo et al. (2014) performed a microarray-based genome-wide analysis of lncRNAs, including 35 LM and MM-matched samples. The authors showed, for the first time, the differential expression profile of the lncRNAs between these tissues. The expression pattern obtained was associated with the downregulation of the cytokine–cytokine receptor interaction pathway in large LM, and the upregulation of the fatty acid metabolism pathway in small LM. This study, although preliminary, sheds light on future studies that will attempt to elucidate the role of lncRNAs specifically in uterine mesenchymal tumors [225].

## 3. Conclusions

Uterine pure sarcomas constitute the most frequently diagnosed group of malignant neoplasms in the uterine body. LMS and ESS are distinct tumors with a variety of features similar to other uterine neoplasms. The high heterogeneity, morphological and molecular variations pose challenges to subtypes differentiation and diagnosis. The origin of these tumors remains unclear, as well as the molecular mechanisms that drive their clinical and biological behavior. However, genetic, and epigenetic mechanisms have been shown to directly and indirectly influence the USMT malignant transformation, but the high complexity of this group of tumors still represents a barrier to diagnosis and disease management. In this review, we provided insights into the most recent studies regarding epigenetic events in LMS and ESS, and their potential as novel biomarkers or for developing new therapeutic modalities to treat these tumors.

## Figures and Tables

**Table 1:** Staging of LMS and ESS (FIGO ^1^ and AJCC ^2^).

Stage	Features	Description
I	T1	Tumor limited to the uterus (T1).
	N0	
	M0	
IA	T1a	Tumor restricted to the uterus (less than 5 cm) (T1a).
	N0	
	M0	
IB	T1b	Tumor restricted to the uterus (more than 5 cm) (T1b).
	N0	
	M0	
II	T2	Tumor growing outside the uterus but is restricted to the pelvis (T2).
	N0	
	M0	
IIIA	T3a	Tumor growing in a single tissue located in the abdomen (T3a).
	N0	
	M0	
IIIB	T3b	Involvement of other extrauterine pelvic tissues, 2 or more sites (T3b).
	N0	
	M0	
IIIC	T1–T3	Tumor invades abdominal tissues (does not protrude from the abdomen) but does not grow into the bladder or rectum (T1 to T3). The cancer has spread to nearby lymph nodes (N1).
	N1	
	M0	
IVA	T4	Tumor spread to the rectum or urinary bladder (T4). It might or might not have spread to nearby lymph nodes (Any N).
	Any N	
	M0	
IVB	Any T	Tumor spread to distant sites (lungs, bones, or liver) (M1). It may or may not have grown into tissues in the pelvis and/or abdomen (any T) and it might or might not have spread to lymph nodes (Any N).
	Any N	
	M1	

^1^ FIGO: International Federation of Gynecology and Obstetrics classification (2009). ^2^ AJCC: American Joint Committee on Cancer TNM staging system (2018).

**Table 2:** Molecular features of endometrial stromal tumors (ESTs).

Category EST	Fusion/Gene Alteration [72,86,87,88,89,90,91,92,93,94,95,96,97,98,99,100,101]
Endometrial Stromal Nodule (ESN)	*JAZF1-SUZ12*^1^ [86,87] *MEAF6-PHF1* [86,87]
Low-Grade Endometrial Stromal Sarcoma (LG-ESS)	*JAZF1-SUZ12*^1^ [88] *JAZF1-PHF1* [88] *MEAF6-PHF1* [88] *EPC1-PHF1* [89] *MBTD1-EZHIP* [89] *JAZF1-BCORL1* [89] *MAGED2-PLAG1* [90] *MEAF6-SUZ12* [91] *EPC2-PHF1* [92] *BRD8-PHF1* [72] *EPC1-BCOR* [72] *EPC1-SUZ12* [72]
High-Grade Endometrial Stromal Sarcoma (HG-ESS)	*YWHAE-NUTM2A/B*^1^ [93] *BCOR*-rearrangement [94] *ZC3H7B-BCOR* [72,95] *EPC1-BCOR* [96] *EPC1-SUZ12* [96] *BCOR-ITD* [72] *LPP-BCOR* [72] *BRD8-PHF1* [97]
Undifferentiated Uterine Sarcoma (UUS)	*JAZF1-SUZ12* [97] *YWHAE-NUTM2* [97] *ZC3H7B-BCOR* [97] *YWHAE*-rearrangement [97] *HMGA-RAD51B* [98] *SMARCA4*-deficient [99]
NTRK-Rearranged Uterine Sarcomas (HG-ESS)	*RBPMS-NTRK3* [100,101] *TPR-NTRK1* [100,101] *LMNA-NTRK1* [100,101] *TPM3-NTRK1* [100,101] *EML4-NTRK3* [100,101] *STRN-NTRK3* [100,101]

^1^ Most common alterations.

## Data Availability

Not applicable.
